# Pressure-induced superconductivity in H_2_-containing hydride PbH_4_(H_2_)_2_

**DOI:** 10.1038/srep16475

**Published:** 2015-11-12

**Authors:** Ya Cheng, Chao Zhang, Tingting Wang, Guohua Zhong, Chunlei Yang, Xiao-Jia Chen, Hai-Qing Lin

**Affiliations:** 1 Shenzhen Institutes of Advanced Technology, Chinese Academy of Sciences and The Chinese University of Hong Kong, Shenzhen, 518055, China; 2 Department of Physics, Yantai University, Yantai, 264005, China; 3 Center for High Pressure Science and Technology Advanced Research, Shanghai 201203, China; 4 Beijing Computational Science Research Center, Beijing 100089, China

## Abstract

High pressure structure, stability, metallization, and superconductivity of PbH_4_(H_2_)_2_, a H_2_-containing compound combining one of the heaviest elements with the lightest element, are investigated by the first-principles calculations. The metallic character is found over the whole studied pressure range, although PbH_4_(H_2_)_2_ is metastable and easily decompose at low pressure. The decomposition pressure point of 133 GPa is predicted above which PbH_4_(H_2_)_2_ is stable both thermodynamically and dynamically with the *C*2/*m* symmetry. Interestedly, all hydrogen atoms pairwise couple into H_2_ quasi-molecules and remain this style up to 400 GPa in the *C*2/*m* structure. At high-pressure, PbH_4_(H_2_)_2_ tends to form the Pb-H_2_ alloy. The superconductivity of *T*_*c*_ firstly rising and then falling is observed in the *C*2/*m* PbH_4_(H_2_)_2_. The maximum of *T*_*c*_ is about 107 K at 230 GPa. The softening of intermediate-frequency phonon induced by more inserted H_2_ molecules is the main origin of the high *T*_*c*_. The results obtained represent a significant step toward the understanding of the high pressure behavior of metallic hydrogen and hydrogen-rich materials, which is helpful for obtaining the higher *T*_*c*_.

In recent decades, many scientists have devote to searching for the high-temperature superconducting materials. For the lightest element, hydrogen (H), Ashcroft applied the BCS theory to propose that the metallic hydrogen will be a room-temperature superconductor under high pressure^1^. This suggestion has motivated considerable experimental and theoretical activities. However, solid hydrogen remains insulating character at extremely high pressure, at least up to 342 GPa^2^. Due to the extremely high and experiment unreachable pressure, as a alternative, Ashcroft proposed that the hydrogen-rich alloys shall transform into metal under relatively lower pressure due to the chemical precompressions from the comparable weight elements^3^. Thus, hydrogen-rich group-IV hydrides have been extensively explored, such as CH_4_, SiH_4_, GeH_4_, SnH_4_, and PbH_4_. All of them show up interesting new structures and novel properties under pressure. CH_4_ is still an insulator up to the pressure of 520 GPa^4^. Although Eremets *et al.* experimentally reported the metallization and superconductivity of SiH_4_ above 60 GPa^5^, for the controversial result it might be understood as superconductivity of amorphous silicon, silicon hydrides, or platinum hydrides^6^,^7^. And theoretical prediction indicates that the stable SiH_4_ can behave as metal and exhibit superconductivity above 220 GPa with the superconducting transition temperature (*T*_*c*_) of about 20 K (The Coulomb parameter 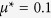, the below is same.)^8^. GeH_4_ has lower metallization pressure than silane^9^,^10^, and the highest *T*_*c*_ reaches to 73 K at 220 GPa^11^. Furthermore, the metallization pressure of SnH_4_ decreases, the highest *T*_*c*_ is close to 83 K at 120 GPa^12^.

It is clearly that the metallization pressure of group-IV hydrides decreases with increase of atomic number of heavy element, which is obviously less than that of solid H_2_. Unfortunately, the *T*_*c*_ of group-IV hydrides is also greatly decreased. By analyzing the crystal feature, we find that the quasi-molecular H_2_ units exist in the high-pressure structures of GeH_4_ and SnH_4_. And these H_2_ units have been found to contribute significantly to the superconductivity. Then, whether can the *T*_*c*_ be improved by intercalating H_2_ into group-IV hydrides? H_2_-containing compounds of CH_4_-H_2_ have been fabricated up to 30 GPa, such as CH_4_(H_2_)_2_, (CH_4_)_2_H_2_, CH_4_(H_2_)_4_, CH_4_H_2_^13^. But both metallization and superconductivity are still lack. For the SiH_4_-H_2_ system, the crystal structure, phase diagram, and metallization under pressure of SiH_4_(H_2_)_2_ were extensively explored^14^,^15^,^16^,^17^,^18^,^19^,^20^,^21^,^22^. The *T*_*c*_ of SiH_4_(H_2_)_2_ is as high as 107 K at 250 GPa^23^, which is visibly higher than that of SiH_4_. Following the experimental observation^24^, we have also theoretically investigated the structural, phase transition, metallization, and superconductivity of GeH_4_(H_2_)_2_ under pressure^25^,^26^. The predicted *T*_*c*_ of GeH_4_(H_2_)_2_ is close to 100 K at 250 GPa, higher than that of GeH_4_. These results inevitably encourage us further to seek for high-temperature superconductors and study the superconductivity in these H_2_-containing compounds. However, it is necessary to decrease the work pressure of superconducting. For examples, the decomposition pressures are as high as 248 GPa for SiH_4_(H_2_)_2_ and 220 GPa for GeH_4_(H_2_)_2_, respectively, above which they are stable superconducting materials.

As mentioned above, the combination the lightest H with one of the heaviest Pb seems to be a good way to improve the *T*_*c*_ and decrease the work pressure. Chemically, PbH_4_ still remains the most elusive of group-IV tetrahydrides. The pioneering theoretical work of Desclaux and Pyykkö predicted the structure and stability of PbH_4_^27^,^28^. The theoretically predicted tetrahedral structure of an isolated molecule, with an equilibrium Pb-H distance of approximately 1.73 Å, was eventually confirmed by experiments^29^,^30^. But, Krivtsun *et al.*^30^ observed that the PbH_4_ molecules were kinetically unstable and readily decompose to Pb atomic layer and H_2_ in approximately 10 s. Recently, Zaleski-Ejgierd *et al.* theoretically investigated the structure and the stability of PbH_4_ under high pressure^31^. They found that PbH_4_ is stable thermodynamically above 132 GPa, in forms of *Imma* (132–296 GPa) and *Ibam* (>296 GPa) space groups. And PbH_4_ even keeps the metallic character covering the whole range of pressure^31^. However, the superconductivity is indeterminate, since the dynamic stable phase of PbH_4_ has been not discovered from experimental and theoretical aspects yet. By intercalating H_2_ units into PbH_4_ molecular crystal, e.g. PbH_4_(H_2_)_2_, how about the structure, stability, and superconductivity? It is just the purpose of our study. In this work, we found out the stable phase of PbH_4_(H_2_)_2_ thermodynamically and dynamically and investigated its desired superconductivity. The decomposition pressure of 133 GPa is much lower than the metallization pressure of solid hydrogen, which is easily reached in experiments by diamond-anvil techniques. And the H_2_-H_2_ coupling under high pressure figures out the different superconducting mechanism.

## Results

**Figure 1:**
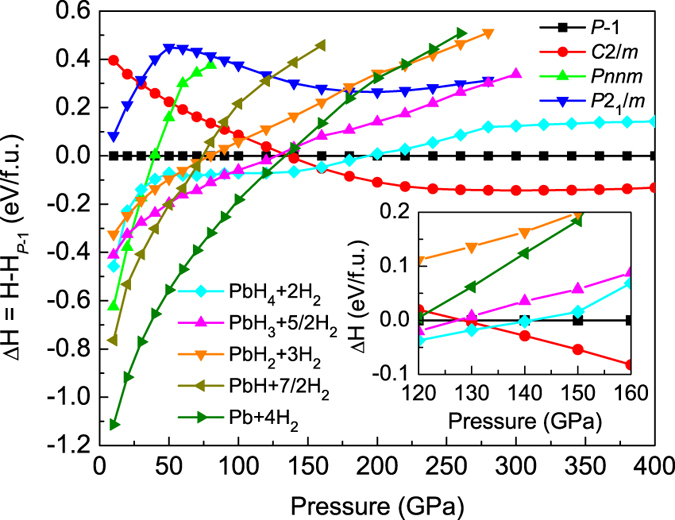
Calculated enthalpies per PbH_4_(H_2_)_2_ unit as the function of pressure. Enthalpy difference versus pressure for competitive structures of PbH_4_(H_2_)_2_, referenced to the *P*-1 phase. The decomposition enthalpies into PbH_4_ + 2H_2_, PbH_3_ + 5/2H_2_, PbH_2_ + 3H_2_, PbH + 7/2H_2_, and Pb + 4H_2_ were also plotted. The inset exhibits the change of enthalpies induced by ZPE correction, which indicates that the decomposition pressure of the *C*2/*m* structure decreases as 133 GPa.

Covering the wide pressure range of 0–400 GPa, variable-cell structure prediction simulations with 1 to 4 PbH_4_(H_2_)_2_ formula units per cell (f.u./cell) were performed. We have calculated the enthalpies of searched structures of PbH_4_(H_2_)_2_ to examine the thermodynamical stability induced by pressure. For several competitive structures of PbH_4_(H_2_)_2_, the enthalpies (relative to the *P*-1 structure) as function of pressure are shown in Fig. 1. It is found that *Pnnm* phase is the most stablest below 40 GPa with the lowest enthalpy value. Starting from 40 GPa up to 135 GPa, PbH_4_(H_2_)_2_ transfers into *P*-1 phase. Upon further compression, the *C*2/*m* becomes to the most stablest phase above 135 GPa. As a result, there are two structural phase transitions existing in the range of 0–400 GPa. Three low-enthalpy structures were obtained, orthorhombic *Pnnm* (4 f.u./cell), triclinic *P*-1 (2 f.u./cell), and monoclinic *C*2/*m* (2 f.u./cell), respectively, as shown in Supplementary Fig. S1 online. The lattice parameters of these three structures at different pressures are also listed in Table S1 of the supplementary information online. From the crystal configurations at different pressures, PbH_4_ tetrahedral molecule does not exist in PbH_4_(H_2_)_2_, and all of hydrogen atoms construct the H_2_ quasi-molecules separating from Pb atoms.

However, it was reported that the hydrogen-rich materials is easily decomposed^10^,^11^,^15^,^16^,^17^,^22^,^23^,^24^,^25^,^31^,^32^. Hence, we must check the stability by mean of estimating the decomposition enthalpy. For PbH_4_(H_2_)_2_, there are five possible decomposition paths as PbH_4_(H_2_)_2_ → Pb + 4H_2_, 2PbH_4_(H_2_)_2_ → 2PbH + 7H_2_, PbH_4_(H_2_)_2_ → PbH_2_ + 3H_2_, 2PbH_4_(H_2_)_2_ → 2PbH_3_ + 5H_2_, and PbH_4_(H_2_)_2_ → PbH_4_ + 2 H_2_, respectively. For three system of PbH_3_, PbH_2_, and PbH, we searched their structures at different pressures. Structural parameters at different pressure regions are presented in Supplementary (Tables S2, S3, and S4) online. With help of the reported structures of *Pmnm*, *P*6/*mmm*, *Imma* and *Ibam* for PbH_4_^31^, *fcc*, *hcp* and 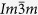 for Pb^33^, *P*6_3_*m*, *C*/2*c*, and *Cmca* for H_2_^34^ corresponding stable pressures, the decomposition enthalpies were calculated and plotted in Fig. 1. PbH_4_(H_2_)_2_ is unstable and decomposes into Pb + 4H_2_ blow 120 GPa and PbH_4_ + 2H_2_ in the pressure range of 120–160 GPa. Namely, both *Pnnm* and *P*-1 phases are metastable. PbH_4_(H_2_)_2_ is only stabilized above the pressure of 160 GPa, displaying the symmetry of *C*2/*m*.

Besides, it has well-known that quantum effects related to hydrogen atoms are very important. The hydrogen zero-point energy (ZPE) has significantly revised the structural stability as in the cases of solid hydrogen^34^ and hydrogen-rich materials^9^,^11^. To judge the effect on stability, we also calculated the ZPEs of PbH_4_(H_2_)_2_, PbH_4_, and H_2_ in the range of 100–200 GPa using the quasiharmonic approximation^35^. As the insert shown in Fig.1, the ZPE effect does not change the order of the phase transitions but lowers the decomposition pressure of the *C*2/*m* structure into ~133 GPa. This decomposition pressure of PbH_4_(H_2_)_2_ is obviously lower than 248 GPa of SiH_4_(H_2_)_2_^23^ and 220 GPa of GeH_4_(H_2_)_2_^25^, which indicates that PbH_4_(H_2_)_2_ will exist in the wider pressure range. For this stability, the subsequent crystal structural, electronic, phonon, and electron-phonon coupling (EPC) calculations are focused on the *C*2/*m* structure above 133 GPa, and typical results are presented at 200 GPa.

**Figure 2:**
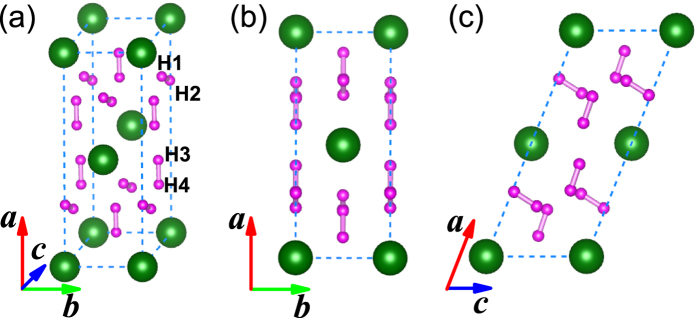
High-pressure crystal structure of PbH_4_(H_2_)_2_. (**a**) *C*2/*m* structure at 200 GPa. Large and small spheres represent Pb and H atoms, respectively. H1-H4 mark four non-equivalent H atoms on the crystallographic sites. (**b**,**c**) show the *C*2/*m* structure normal to the (001) and (010) plane, respectively.

**Figure 3:**
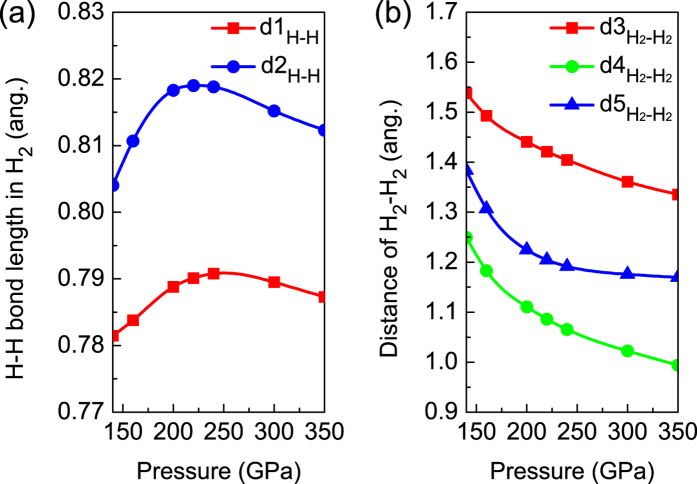
The H-H bond lengths in H_2_ unit and the H_2_-H_2_ intermolecular distances. For *C*2/*m* structure, two types of H-H bond lengths in H_2_ (**a**) and three kinds of distances among H_2_ molecules (**b**) change with pressure (133–350 GPa).

For *C*2/*m* structure, Pb atoms occupy the crystallographic 2*a* sites and four non-equivalent H atoms sit on the 4*i* sites under high pressure. All of H atoms pairwise coupling into two types of quasi-molecules as shown in Fig. 2a. The nearest distance between Pb and H atom is about 2 Å. In this dense structure, we can not find any plumbane molecules existing, but H_2_ quasi-molecules distribute around Pb atoms and are ordering (Fig. 2). This kind of ordered arrangements of H_2_ units is clearer at high pressure, while H_2_ units tend to be inordering at low pressure^18^,^25^. A visible character of Pb and H_2_ in layers is observed from (001)-plane (Fig. 2b) or (010)-plane (Fig. 2c). Noticeably, the layered feature is also a common phenomenon in some hydrogen-rich systems. With the increase of pressure, all of the lattice constants of *C*2/*m* structure in *a*, *b*, and *c* directions decrease. However, the H-H bond lengths in H_2_ quasi-molecules marked as d1_*H*−*H*_ (formed by H1 and H2 sites shown in Fig. 2a) and d2_*H*−*H*_ (formed by H3 and H4 sites shown in Fig. 2a) firstly increase then decrease as shown in Fig. 3a. There are three kinds of intermolecular distances among H_2_ molecules in the *C*2/*m* structure, all of them are monotonously decreased with the pressurizing, as shown in Fig. 3b. Reviewing the high-pressure structural character, we find that part hydrogen atoms form H_2_ units with the other hydrogen atoms strongly bonding with Si in *Ccca* phase of SiH_4_(H_2_)_2_^23^, while all of hydrogen atoms pairwise coupling into H_2_ quasi-molecules with the nearest distance of ~1.7 Å between Ge and H in *P*2_1_/*c* phase of GeH_4_(H_2_)_2_^25^. As a comparison, with the help of analysis of atomic distances, the intermolecular and intramolecular couplings of H_2_ gradually strengthen, while the interaction between H and the heavy atom evidently weakens from SiH_4_(H_2_)_2_ to GeH_4_(H_2_)_2_ and then to PbH_4_(H_2_)_2_.

**Figure 4:**
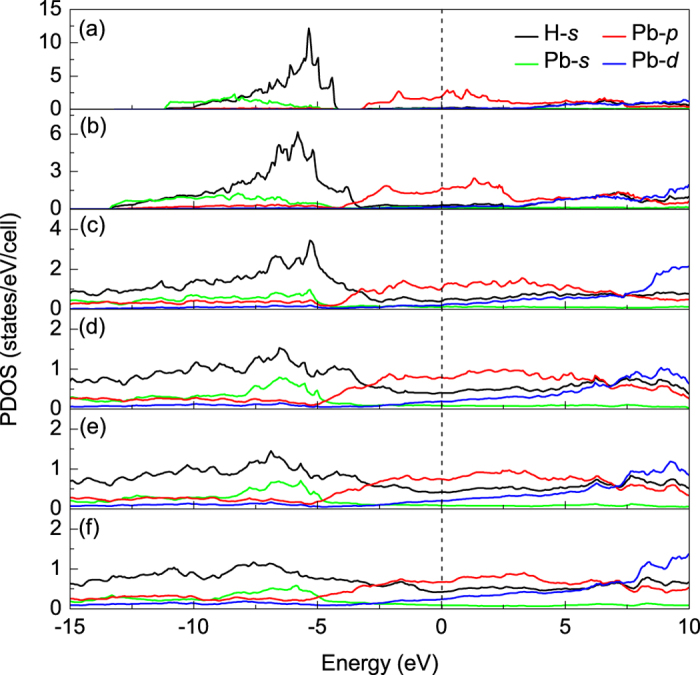
Electronic PDOS at different pressures. Calculated PDOS of PbH_4_(H_2_)_2_ at different pressures of 5 GPa (**a**) and 20 GPa (**b**) for *P*-1 phase, 100 GPa for *Pnnm* phase (**c**), 160 GPa (**d**), 200 GPa (**e**), and 300 GPa (**f**) for *C*2/*m* phase. The lines at zero indicate the Fermi level.

**Figure 5:**
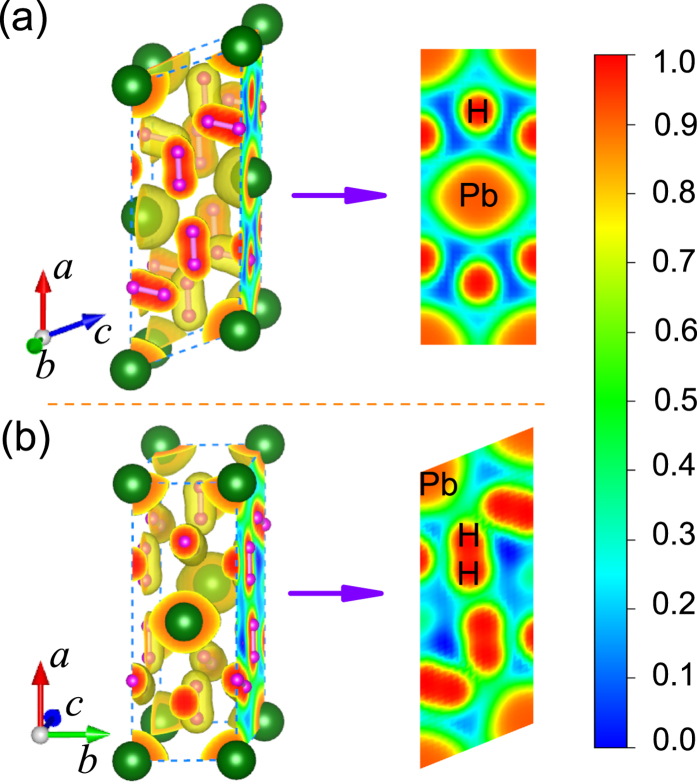
ELF of PbH_4_(H_2_)_2_. Calculated ELF isosurface of PbH_4_(H_2_)_2_ for *C*2/*m* at 200 GPa with the ELF value of 0.75. (**a**,**b**) highlight the sections on (001) and (010) planes, respectively.

At 200 GPa, the lattice parameters of *C*2/*m* structure are *a* = 7.184 Å, *b* = 2.807 Å, and *c* = 2.973 Å, as well as the angle *β* = 68.1° (see Supplementary Table S1 online). The d1_*H*−*H*_ and d2_*H*−*H*_ are 0.78 Å and 0.82 Å, respectively. The intermolecular distance of H_2_-H_2_ is less than that between Pb and H atoms. With the lattice parameters, calculated electronic structures show that PbH_4_(H_2_)_2_ is metallic at 200 GPa. For SiH_4_(H_2_)_2_ and GeH_4_(H_2_)_2_ reported previously, they remain the characteristics of insulator under low pressure. The insulator-to-metal transition occurs at 92 GPa in SiH_4_(H_2_)_2_ and at 48 GPa in GeH_4_(H_2_)_2_, respectively. However, we didn’t find the transition point of PbH_4_(H_2_)_2_. It seems to be metal even in ambient pressure, which consist with PbH_4_^31^. So the low pressure metallization does not come from the intercalation of H_2_ molecules. Comparing with Si and Ge, Pb has larger ionic radius which results in more strong itinerant property of valent electrons. Figure 4 shows the projected density of state (PDOS) at several selected pressures. According to the electronic PDOS at Fermi level we can draw a conclusion that at low pressure in *Pnnm* structure the Pb-*p* electrons make the most contribution to density of state and exhibit properties of a nearly free-electron metal (Fig. 4a,b). As the pressure increases, the strengthening of H_2_-H_2_ interaction leads to the overlap of H-*s* wave functions. The contribution of H-*s* electrons to Fermi surface increases. PDOS tends to be uniform distribution, and the bandwidth further broadens from 100 GPa to 300 GPa (Fig. 4c–f). It indicates that with the increase of pressure PbH_4_(H_2_)_2_ mainly like to be Pb-H_2_ alloy. The Pb interlayer interaction is connected by these H_2_ molecules. To gain more insight into the bonding nature of PbH_4_(H_2_)_2_, the electron location function (ELF) of *C*2/*m* phase at 200 GPa was calculated. ELF shown in Fig. 5 displays the electronic location around Pb and H atoms as well as the nearly free-electron-like distribution among Pb atoms. However, the high ELF values between Pb and H atoms (Fig. 5a) and of intermolecular H_2_ (Fig. 5b) indicate that the electrons become delocalized, suggesting a feature of nearly free-electron metal.

**Figure 6:**
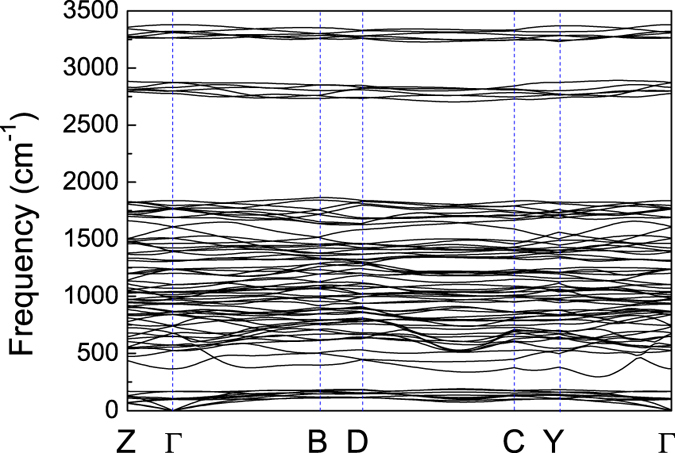
Phonon spectrum. Calculated phonon spectrum of *C*2/*m* structure at 200 GPa.

**Figure 7:**
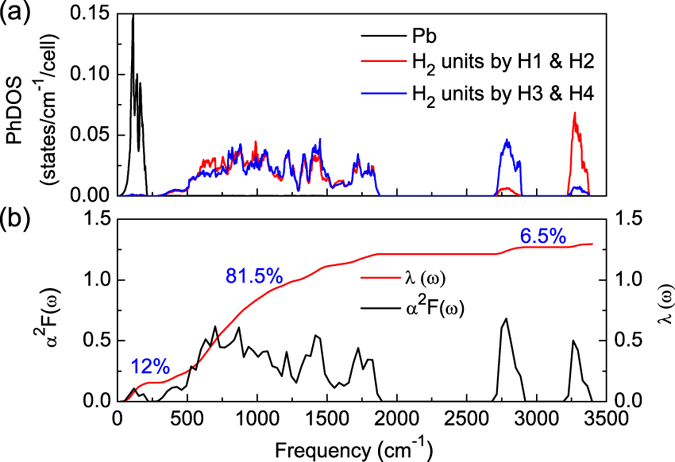
Phonon properties and Eliashberg spectral function. Calculated phonon density of states (PhDOS) (**a**), and the Eliashberg phonon spectral function *α*^2^*F*(*ω*) and electron-phonon integral *λ*(*ω*) (**b**) for the *C*2/*m* structure at 200 GPa.

The phonon dispersion curves for *C*2/*m* structure at 200 GPa (Fig. 6) and other selected pressure point (see Supplementary Fig. S2 online) were calculated to explore the lattice dynamics of PbH_4_(H_2_)_2_. The absence of any imaginary frequencies implies the dynamical stability of *C*2/*m* phase under high pressure. The whole phonon spectrum can be divided into three parts. By combining with the phonon density of states (PhDOS) projected on atoms shown in Fig. 7a, in the case of 200 GPa, we find that the low-frequency vibration below 215 cm^−1^ mainly come from the vibrations Pb atoms. The intermolecular strong phonon coupling among H_2_ molecules appear in the intermediate-frequency range of 295–1876 cm^−1^. After a large gap, in high frequency area above 2695 cm^−1^, the H-H vibration in H_2_ formed by H3 and H4 sites mainly contributes in the range of 2695–2898 cm^−1^, while the vibration in H_2_ formed by H1 and H2 sites around 3220 to 3380 cm^−1^. Comparing these three systems of Si-, Ge-, and Pb-based, we find a strong phonon coupling between silicon and hydrogen in SiH_4_(H_2_)_2_^23^, very weak phonon coupling between metal and hydrogen in GeH_4_(H_2_)_2_^25^ as well as PbH_4_(H_2_)_2_. The H-H vibration in H_2_ molecule is the strongest in PbH_4_(H_2_)_2_. From the Eliashberg phonon spectral function *α*^2^*F*(*ω*) and the integrated EPC parameter *λ*(*ω*) shown in Fig. 7b, the intermediate-frequency (295–1876 cm^−1^) vibrational modes of H_2_ molecules contribute 81.5% of total *λ*. This percentage is larger than 66% in Si-based and 75% in Ge-based case. This result highlights the significant role played by H_2_ molecules on the electron-phonon interaction.

At 200 GPa, the calculated total EPC constant *λ* is 1.296 for *C*2/*m* PbH_4_(H_2_)_2_. From Si to Ge and then to Pb case, the *λ* gradually decreases from 1.625 to 1.43 and then to 1.296, which implies a weak coupling between metal and hydrogen. However, the phonon frequency logarithmic average *ω*_*log*_ rises gradually, from 871 K in SiH_4_(H_2_)_2_ to 1051 K in PbH_4_(H_2_)_2_. This means more higher Debye temperature in PbH_4_(H_2_)_2_. Based on the obtained *α*^2^*F*(*ω*) and *λ*(*ω*), we now can analyze the superconductivity using the modified McMillan equation by Allen and Dynes^36^,


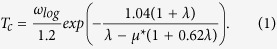


With the typical choice of the Coulomb pseudopotential 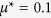
3, a remarkable large *T*_*c*_ of 103 K was obtained for *C*2/*m* phase of PbH_4_(H_2_)_2_, which is comparable with those of copper oxide superconductors.

To figure out the pressure effect on superconductivity in PbH_4_(H_2_)_2_, in addition, the *T*_*c*_ values at several typical pressure points were calculated and shown in Supplementary Fig. S3 online. An interesting phenomenon exhibits the superconductivity firstly strengthening before weakening. The *T*_*c*_ has a maximum between 140 and 350 GPa, ~107 K for 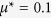. Seen from the distances of among H_2_ molecules shown in Fig. 3b, the monotonously decreasing makes a hint of “hardening” of intermediate-frequency phonon with the increase of pressure. The phonon spectra shown in Supplementary Fig. S2 online confirm this point. To analyze this phenomenon of *T*_*c*_ variations, we have further calculated the Eliashberg phonon spectral function and the EPC strength at different pressures, the results are presented in Supplementary Fig. S4 online. With the increase of pressure, the calculated EPCs are 1.280, 1.296, 1.379, and 1.341 for 180 GPa, 200 GPa, 250 GPa, and 300 GPa, respectively, which shows a tendency of first increase and then decrease similar to *T*_*c*_. In the *T*_*c*_ rising zone, the contribution of Pb-H coupling to the EPC strength is decreased from 14.3% at 180 GPa to 12% at 200 GPa, and the phonon vibration of H-H in H_2_ units also weakens the EPC (The contribution is from 7.3% to 6.5% corresponding pressures.). However, the contribution of H_2_-H_2_ coupling to the EPC is strengthening from 78.4% at 180 GPa to 81.5% at 200 GPa. So the initial rising of *T*_*c*_ results from the contribution increasing of H_2_-H_2_ for the EPC. As shown in Fig. S4 online, from 75.8% at 250 GPa to 73.5% at 300 GPa, the decrease of contribution of H_2_-H_2_ for the EPC leads to the fall of *T*_*c*_. The result further reveals the significance of H_2_-H_2_ coupling to superconductivity in PbH_4_(H_2_)_2_.

## Discussion

Thus far, the stability of PbH_4_(H_2_)_2_ has been identified in the pressure range of 0–400 GPa. At low pressure it is metastable and possibly decomposes into Pb + H_2_ or PbH_4_ + H_2_. Above 133 GPa, it is stable not only thermodynamically but also dynamically. This high-pressure stable phase of *C*2/*m* exhibits the expected superconductivity of *T*_*c*_ ~ 107 K at 230 GPa, which is obviously higher than those of conventional group-IV hydrides such as silane, germane, and stannane. Noticeably, the coupling between group-IV element and hydrogen reduces with the increase of atomic number. Namely, the contribution of group-IV element to total EPC decreases in hydrides from 33% in SiH_4_(H_2_)_2_^23^ to 25% in GeH_4_(H_2_)_2_^25^ and then to 12% in PbH_4_(H_2_)_2_. On the contrary, the coupling among H_2_ molecules strengthens as mentioned above. Particularly, we want to point out that the *T*_*c*_ (~100 K) is comparable for SiH_4_(H_2_)_2_, GeH_4_(H_2_)_2_, and PbH_4_(H_2_)_2_ at the same Coulomb pseudopotential, though the superconducting mechanism is incompletely same. The intercalating H_2_ molecules into group-IV hydrides really improves the *T*_*c*_. From the phonon contribution to EPC, we find that the intermediate-frequency phonon is dominated. Comparing with corresponding SiH_4_^8^, GeH_4_^11^, and SnH_4_^12^, it is clear that the intercalation of H_2_ molecules results in the softening of intermediate-frequency phonon. As increasing the content of hydrogen in group-IV elements, it results in enhancing the EPC strength that is dominated by the coupling of the H_2_ molecular in the *A*H_4_(H_2_)_2_ (*A* = Si, Ge, Sn, and Pb) crystals. This is just the origin of higher *T*_*c*_ in H_2_-containing compounds. Furthermore, we infer that the higher *T*_*c*_ may be obtained if more H_2_ are inserted in group-IV hydrides. Actually, more future works are needed to advance the *T*_*c*_ and understand the superconductivity.

As a comparison, the high-pressure structure of PbH_4_(H_2_)_2_ is visibly different from other hydrogen-rich compounds with high *T*_*c*_, such as CaH_6_^37^ and (H_2_S)_2_H_2_^32^. In high-pressure structures of CaH_6_ and (H_2_S)_2_H_2_, the H_2_ quasi-molecules have been broken, with the strong bonds forming between metal and hydrogen atoms. Although the EPC is mainly contributed by hydrogen, the superconducting mechanism is different. It is the H-H coupling in CaH_6_ and (H_2_S)_2_H_2_, while the H_2_-H_2_ coupling in PbH_4_(H_2_)_2_. It is interested that the H_2_ quasi-molecule form keeps all along at thus high pressure up to 400 GPa. At the same time, Pb is one of the heaviest elements. The combination with the lightest H is one of the most important physical problems in high-pressure research. Pb metal makes the metallization pressure of hydrogen-rich compound decrease. Remarkably, the decomposition pressure point (133 GPa) of PbH_4_(H_2_)_2_ is the lowest among these H_2_-containing compounds of Si-, Ge-, and Pb-based. This value is much lower than the metallization pressure of bulk molecular hydrogen, which indicates the feasibility to experimentally observe. Hence, Pb-based hydrides are the potential candidates as high-*T*_*c*_ superconductors. Our finding may hopefully stimulate the potential high-*T*_*c*_ superconductors research in H_2_-containing hydrides.

## Methods

The search for crystalline structures of PbH_4_(H_2_)_2_ phases was performed using particle swarm optimization methodology as implemented in the CALYPSO program^38^,^39^. Structural optimizations, enthalpies, and electronic structures were calculated using the Vienna ab initio simulation (VASP) program^40^,^41^ and projector-augmented plane wave (PAW) potentials employing the Perdew-Burke-Ernzerhof (PBE) functional^42^. The 1*s*^1^ and 6*s*^2^6*p*^2^ electrons were included in the valence space for H and Pb atoms, respectively. For the plane-wave basis-set expansion, an energy cutoff of 800 eV was used. Dense *k*-point meshes were employed to sample the first Brillouin zone (BZ) and ensured that energies converged to within 1 meV/atom. All forces acting on atoms were converged 0.001 eV/Å or less, and the total stress tensor was reduced to the order of 0.01 GPa. With the noteworthy mass ratio 207:1 between Pb and H, we have involved the spin-orbit effect in this calculation.

Based on the optimized structures from VASP, lattice dynamics and superconducting properties were calculated using density functional perturbation theory^43^ and the Troullier-Martins norm-conserving potentials^44^, as implemented in the QUANTUMESPRESSO code^45^. The cutoff energies of 60 and 400 Ry were used for wave functions and charge densities, respectively. 12 × 12 × 8 Monkhorst-Pack *k*-point grid with Gaussian smearing of 0.03 Ry was used for the phonon calculations at 3 × 3 × 2 *q*-point mesh, and double *k*-point grid was used in the calculation of the electron-phonon interaction matrix element.

## Additional Information

**How to cite this article**: Cheng, Y. *et al.* Pressure-induced superconductivity in H_2_-containing hydride PbH_4_(H_2_)_2_. *Sci. Rep.* **5**, 16475; doi: 10.1038/srep16475 (2015).

## Supplementary Material

Supplementary Information
