# Supplementary material for: Pressure-induced superconductivity in H2-containing hydride PbH4(H2)2
Source: Sci Rep. 2015 Nov 12;5:16475. doi: 10.1038/srep16475 (PMC4642309; doi:10.1038/srep16475)
Supplement: Supplementary Information [file srep16475-s1.pdf]

Supplementary information

Pressure-induced superconductivity in H_2-containing hydriden $PbH_4(H_2)_2$

Ya Cheng,[1] Chao Zhang,[2] Tingting Wang,[1] Guohua Zhong,[1,*] Chunlei Yang[1,†], Xiao-Jia Chen[3] and Hai-Qing Lin[4,1]

[1]Shenzhen Institutes of Advanced Technology, Chinese Academy of Sciences and The Chinese University of Hong Kong, Shenzhen 518055, China
[2]Department of Physics, Yantai University, Yantai, 264005, China
[3]Center for High Pressure Science and Technology Advanced Research, Shanghai 201203, China
[4]Beijing Computational Science Research Center, Beijing 100089, China

Table S1. Lattice parameters and atomic coordinates of $PbH_4(H_2)_2$ at difference pressures.

space group	Pressure (GPa)	Lattice parameters (Å, deg)	atom	x	y	z
Pnnm	20	a=4.820	Pb1(2c)	0.00000	0.50000	-1.00000
		b=13.626	Pb2(2a)	0.00000	0.00000	-1.00000
		c=3.512	H1(4g)	0.48622	0.70157	-1.00000
		α=β=γ=90	H2(4g)	0.51304	0.35404	-1.00000
			H3(4g)	0.76642	0.20436	-1.00000
			H4(4g)	0.70258	0.85072	-1.00000
			H5(4g)	0.26227	0.20350	-1.00000
			H6(4g)	0.00796	0.28016	-1.00000
			H7(4g)	0.52301	0.16424	-1.00000
			H8(4g)	0.22661	0.34951	-1.00000
P-1	160	a=2.896<br>b=4.161<br>c=5.629<br>α=71.0<br>β=88.2<br>γ=70.8	Pb1 (2i)	1.16788	0.14745	-0.84911
			H1 (2i)	2.54257	-0.52858	-1.26204
			H2 (2i)	4.86030	-1.30130	-1.75250
			H3 (2i)	0.61711	0.29962	-0.55204
			H4 (2i)	0.64295	0.30721	-0.41449
			H5 (2i)	3.04141	-0.54666	-1.37609
			H6 (2i)	0.55130	0.42805	-0.11390
			H7 (2i)	1.26202	-0.00233	-0.41815
			H8 (2i)	1.18245	0.19620	-0.51149
*C*2/*m*	200	a=7.184<br>b=2.807<br>c=2.973<br>α=γ=90<br>β=68.1	Pb1(2a)	0.00000	0.00000	0.00000
			H1(4i)	-0.77734	0.00000	2.30255
			H2(4i)	-0.79226	0.50000	2.42086
			H3(4i)	-0.78633	-0.50000	0.97864
			H4(4i)	-0.90858	-0.50000	1.43768

Table S2. Lattice parameters and atomic coordinates of PbH_3 at special pressures.

space group	Pressure (GPa)	Lattice parameters (Å, deg)	atom	x	y	z
P4/mmm	30	a=4.015	Pb1(1a)	0.00000	0.00000	0.00000
(<45 GPa)		b=4.015	Pb2(1c)	0.50000	0.50000	0.00000
		c=4.422	H1(2h)	0.50000	0.50000	0.41369
		α=β=γ=90	H2(4m)	0.40154	0.00000	0.50000
C2/c	80	a=4.416<br>b=8.505	Pb1 (4a)	0.00000	0.00000	0.00000
(45-110		c=5.543	Pb2 (4e)	0.00000	0.32201	0.25000
GPa)		α=90<br>β=91.4	H1 (4c)	0.25000	0.25000	0.00000
		γ=90	H2 (4b)	0.00000	0.50000	0.00000
			H3 (4d)	0.25000	0.25000	0.50000
			H4 (4e)	0.00000	0.68690	0.25000
I4/mmm	150	a=3.366<br>b=3.366	Pb1(2b)	0.00000	0.00000	0.50000
(>110 GPa)		c=3.772	H1(2a)	0.00000	0.00000	0.00000
		α=β=γ=90	H2(4d)	0.00000	0.50000	0.25000

Table S3. Lattice parameters and atomic coordinates of PbH_2 at special pressures.

space group	Pressure (GPa)	Lattice parameters (Å, deg)	atom	x	y	z
I4/mcm (<68 GPa)	30	a=4.070<br>b=4.070<br>c=15.041<br>α=β=γ=90	Pb1(8g)	0.00000	0.50000	0.84400
			H1(32m)	0.29940	0.20060	0.97530
Cm (68-140 GPa)	100	a=3.724<br>b=9.530<br>c=3.936<br>α=90<br>β=93.9<br>γ=90	Pb1 (2a)	0.00025	0.00000	0.03751
			Pb2 (4b)	0.50005	0.14877	0.49025
			H1 (2a)	0.00030	0.00000	0.56036
			H2 (4b)	0.50002	0.24996	0.98796
			H3 (4b)	0.74994	0.75004	0.97751
			H4 (2a)	0.49945	0.00000	0.87817
Cmmm (>140 GPa)	150	a=3.937<br>b=3.638<br>c=2.725<br>α=β=γ=90	Pb1(2c)	0.00000	0.50000	0.50000
			H1(4g)	0.39948	0.50000	0.00000

Table S4. Lattice parameters and atomic coordinates of PbH at special pressures.

space group	Pressure (GPa)	Lattice parameters (Å, deg)	atom	x	y	z
*C*2 (<32 GPa)	20	a=5.590	Pb1(4c)	0.75459	0.91455	0.64315
		b=9.214	Pb2(2a)	0.00000	0.16874	0.00000
		c=3.092	H1(2b)	0.00000	0.66666	0.50000
		α=γ=90	H2(4c)	0.00303	0.66775	0.16443
		β=93.3				
P4/nmm (32-97 GPa)	60	a=2.901<br>b=2.901<br>c=4.952<br>α=β=γ=90	Pb (2c)<br>H (2c)	0.00000<br>0.00000	0. 50000<br>0.50000	0.72817<br>0.12580
Cmcm (>97 GPa)	150	a=4.235<br>b=4.281<br>c=3.925<br>α=β=γ=90	Pb(4c)<br>H(4c)	0.00000<br>0.00000	0.77521<br>0.30866	0.75000<br>0.75000

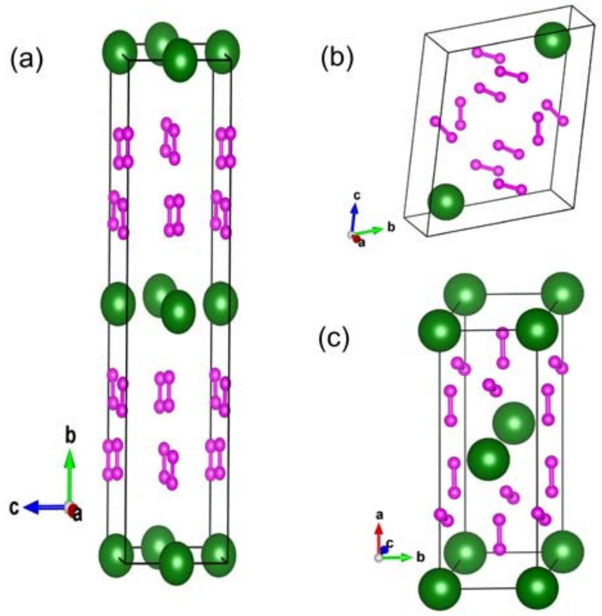

Fig. S1 **Structures of $PbH_4(H_2)_2$.** (a) *Pnnm* structure at 20 GPa, (b) *P*-1 structure at 100 GPa, (c) *C*2/*m* structure at 200 GPa. Large and small spheres represent Pb and H atoms, respectively.

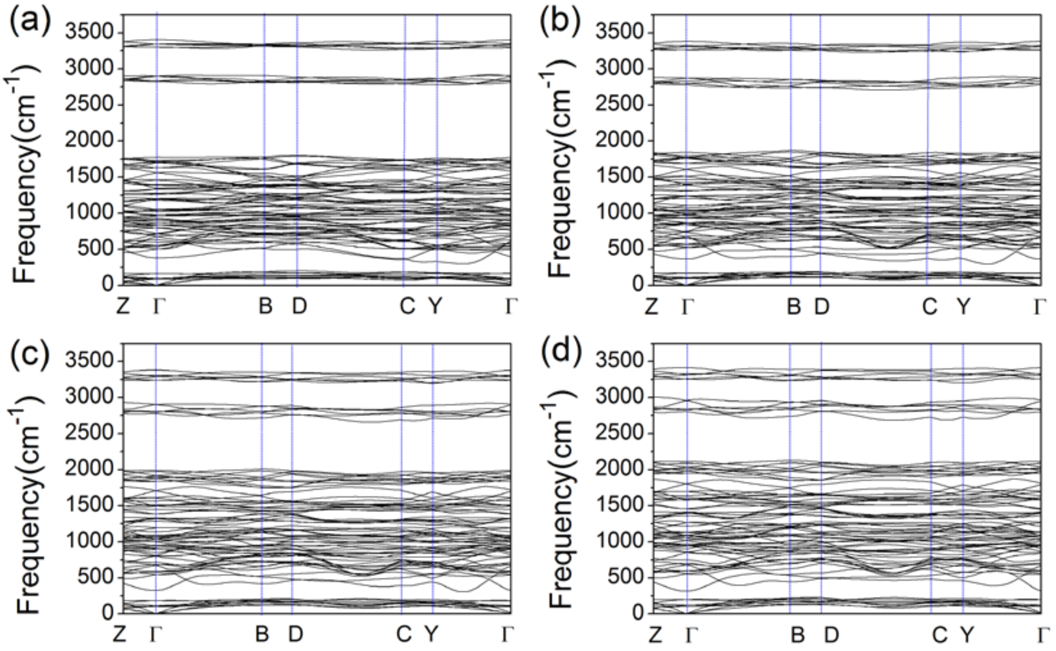

Fig. S2 **Phonon spectra.** Calculated phonon frequencies of *C*2/*m* at 180 GPa (a), 200 GPa (b), 250 GPa (c), and 300 GPa (d), respectively.

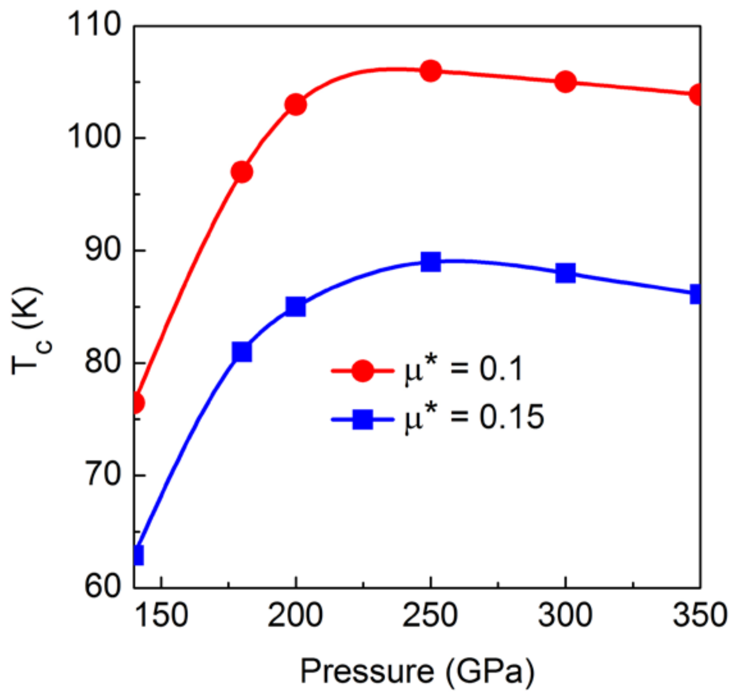

Fig. S3 **Pressure effect of T_c.** Calculated T_c dependence on pressure for *C*2/*m* phase in the range of 133-350 GPa.

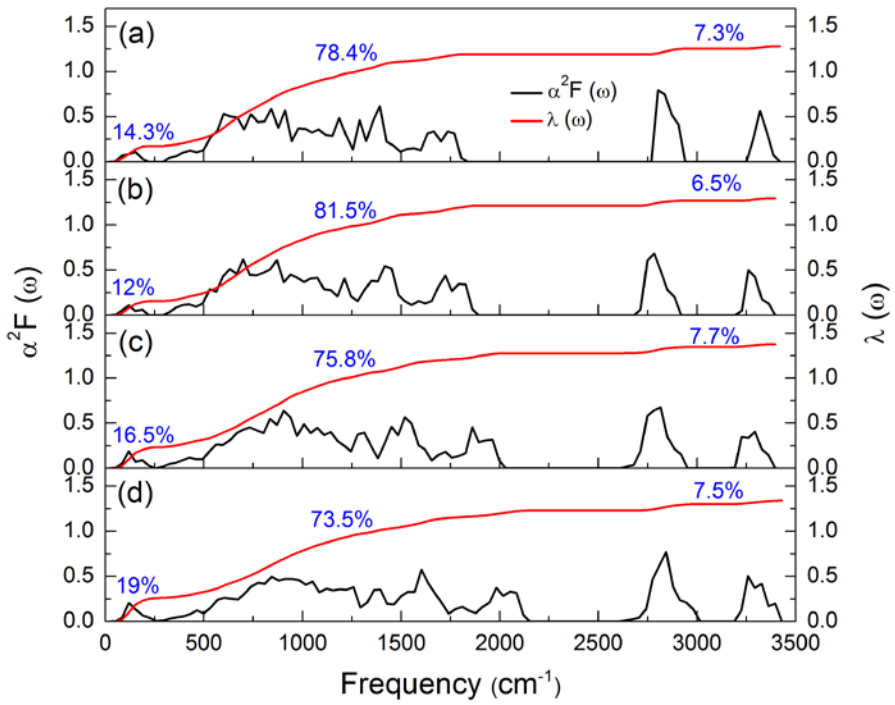

Fig. S4 **Phonon properties and Eliashberg spectral function.** Calculated Eliashberg phonon spectral function $\alpha^2F(\omega)$ and electron-phonon integral $\lambda(\omega)$ for the *C*2/*m* structure. (a) at 180 GPa, λ=1.280, (b) at 200 GPa, λ=1.296, (c) at 250 GPa, λ=1.379, (d) at 300 GPa, λ=1.341.
